# Supplementary material for: Sox9 prevents retinal degeneration and is required for limbal stem cell differentiation in the adult mouse eye
Source: eLife. 2025 Nov 5;13:RP102337. doi: 10.7554/eLife.102337 (PMC12588606; doi:10.7554/eLife.102337)
Supplement: MDAR checklist [file elife-102337-mdarchecklist1.pdf]

Materials Design Analysis Reporting (MDAR) Checklist for Authors

The MDAR framework establishes a minimum set of requirements in transparent reporting mainly applicable to studies in the life sciences.

eLife asks authors to **provide detailed information within their article** to facilitate the interpretation and replication of their work. Authors can also upload supporting materials to comply with relevant reporting guidelines for health-related research (see EQUATOR Network), life science research (see the BioSharing Information Resource), or animal research (see the ARRIVE Guidelines and the STRANGE Framework; for details, see *eLife*'s Journal Policies). Where applicable, authors should refer to any relevant reporting standards materials in this form.

For all that apply, please note **where in the article** the information is provided. Please note that we also collect information about data availability and ethics in the submission form.

Materials:

Newly created materials	Indicate where provided: section/figure legend	N/A
The manuscript includes a dedicated "materials availability statement" providing transparent disclosure about availability of newly created materials including details on how materials can be accessed and describing any restrictions on access.	No newly created biological materials (e.g., novel transgenic lines, plasmids, or cell lines) were generated in this study. All mouse lines used were obtained from established repositories: Sox9^flox/flox^ (Jackson Laboratory, RRID:IMSR_JAX:013106), CAGG-CreER^TM^ (Jackson Laboratory), R26R-LacZ (Jackson Laboratory, RRID:IMSR_JAX:003474), R26R-EYFP (Jackson Laboratory, RRID:IMSR_JAX:006148), and Sox9^IRES-CreERT2^ (RIKEN BioResource Research Center). Experimental animals generated by crossing these lines are available from the corresponding authors upon reasonable request.	X

Antibodies	Indicate where provided: section/figure legend	N/A
For commercial reagents, provide supplier name, catalogue number and RRID, if available.	Histology and immunofluorescence:<br><br>**Primary antibodies:**<br><br>• anti-SOX8 (1:500) - Dr.	

	Michael Wegner, Universität Erlangen-Nürnberg, Germany - RRID:AB_2571872<br>• anti-OPN1SW (1:200) - Santa Cruz Biotechnology, sc-14363 - RRID:AB_2158332<br>• anti-OPN1LW (1:500) - Chemicon/Millipore, AB5405 - RRID:AB_177456<br>• anti-RHO (1:500) - Sigma-Aldrich, O4886 - RRID:AB_258585<br>• anti-BRN3 (1:100) - Chemicon/Millipore, MAB1585 - RRID:AB_94166<br>• anti-PAX6 (1:40) - Developmental Studies Hybridoma Bank (DSHB), University of Iowa - RRID:AB_528427<br>• anti-AP2 (1:50) - Abcam, ab108311 - RRID:AB_10862197<br>• anti-SOX9 (1:400) - Millipore, AB5535 - RRID:AB_2239761<br>• anti-GFP (1:100) - Novus Biologicals, NB600-308 - RRID:AB_10003058<br>• anti-p63 (1:500) - Master Diagnostica/Vitro, MAD-000479QD - **RRID not available**<br>• anti-S100 (prediluted 1:1) - Master Diagnostica/Vitro, MAD-000592Q - **RRID not available**<br>**Note:** The anti-p63 and anti-S100 antibodies from Master	

	Diagnostica (now Vitro Bio) are commercial IVD (in vitro diagnostic) certified reagents but do not have assigned RRIDs in the Antibody Registry. These are proprietary diagnostic antibodies with catalog numbers MAD-000479QD and MAD-000592Q respectively.	

DNA and RNA sequences	Indicate where provided: section/figure legend	N/A
Short novel DNA or RNA including primers, probes: Sequences should be included or deposited in a public repository.	This study did not report the use of novel DNA or RNA sequences, primers, or probes. Mouse genotyping was performed using standard PCR protocols for commercially available transgenic lines (Sox9^flox/flox^, CAGG-CreER^TM^, R26R-LacZ, R26R-EYFP, and Sox9^IRES-CreERT2^) following supplier recommendations. No custom-designed oligonucleotides or novel genetic constructs were generated or required for the experimental procedures described.	X

Cell materials	Indicate where provided: section/figure legend	N/A
Cell lines: Provide species information, strain. Provide accession number in repository OR supplier name, catalog number, clone number, OR RRID.	No cell lines or primary cell cultures were used in this study. All experiments were conducted in vivo using intact mouse tissues (retina and cornea) or ex vivo using freshly dissected tissues processed for histological and immunofluorescence analyses. No cultured cells, immortalized cell lines, or primary cell cultures were established or utilized.	X
Primary cultures: Provide species, strain, sex of origin, genetic modification status.	No primary cell cultures were established or used in this study. All experimental work was performed in vivo using intact mouse tissues or ex vivo using	X

	freshly dissected tissues processed directly for histological, immunofluorescence, or whole-mount analyses without prior cell culture. The scRNA-seq dataset analyzed was obtained from a publicly available repository (Gene Expression Omnibus GSE167992, from Altshuler et al., 2021) and no primary cultures were generated for this dataset by the authors of the present manuscript.	

**Experimental animals**	**Indicate where provided: section/figure legend**	**N/A**
Laboratory animals or Model organisms: Provide species, strain, sex, age, genetic modification status. Provide accession number in repository OR supplier name, catalog number, clone number, OR RRID.	Mouse lines and crosses:<br><br>**Species:** *Mus musculus* (laboratory mouse)<br><br>• **Age:** 2 months old at the start of experiments (adult mice)<br>• **Strains and genetic modifications used:**<br>• Sox9^flox/flox^ (B6.129S7-Sox9^tm2Crm^/J) - The Jackson Laboratory - RRID:IMSR_JAX:013106<br>• CAGG-CreER^TM^ (B6.Cg-Tg(CAG-cre/Esr1)^5Amc^) - The Jackson Laboratory - RRID:IMSR_JAX:004682<br>• R26R-LacZ (B6.129S4-Gt(ROSA)26Sor^tm1Sor^/J) - The Jackson Laboratory -	

	RRID:IMSR_JAX:003474<br>• R26R-EYFP (B6.129X1-Gt(ROSA)26Sor^tm1(EYFP)Cos^/J) - The Jackson Laboratory - RRID:IMSR_JAX:006148<br>• Sox9^IRES-CreERT2^ (B6.129S7-Sox9^tm1(cre/ERT2)Haak^) - RIKEN BioResource Research Center, Tsukuba, Japan - RRID:IMSR_RBRC05522	
Animal observed in or captured from the field: Provide species, sex, and age where possible.	All animals used in this study were laboratory-bred mice housed under Specific Pathogen-Free (SPF) conditions at the Center for Biomedical Research, University of Granada, Spain. No wild-caught or field-observed animals were used.	X

**Plants and microbes**	**Indicate where provided: section/figure legend**	**N/A**
Plants: provide species and strain, ecotype and cultivar where relevant, unique accession number if available, and source (including location for collected wild specimens).	No plant species or materials were used in this study.	X
Microbes: provide species and strain, unique accession number if available, and source.	No microbial species, cultures, or pathogens were used in this study. All experiments were conducted using mouse tissues and commercially available reagents.	X

**Human research participants**	**Indicate where provided: section/figure legend) or state if these demographics were not**	**N/A**

	collected	
If collected and within the bounds of privacy constraints report on age, sex, gender and ethnicity for all study participants.	No human participants or human-derived samples were used in this study. All experiments were conducted using laboratory mice (*Mus musculus*).	**X**

Design:

Study protocol	Indicate where provided: section/figure legend	N/A
If the study protocol has been pre-registered, provide DOI. For clinical trials, provide the trial registration number OR cite DOI.	This study was not pre-registered. No clinical trials were conducted.	X

Laboratory protocol	Indicate where provided: section/figure legend	N/A
Provide DOI OR other citation details if detailed step-by-step protocols are available.	Detailed protocols were not deposited in external repositories. All methodological details are provided in the Material and Methods section of the manuscript.	X

Experimental study design (statistics details) *		
**For in vivo studies: State whether and how the following have been done**	**Indicate where provided: section/figure legend. If it could have been done, but was not, write "not done"**	**N/A**
Sample size determination	not done	
Randomisation	not done	
Blinding	not done	
Inclusion/exclusion criteria	All mice analyzed in the study received tamoxifen treatment to induce Cre-mediated recombination. Control animals were those that received tamoxifen but showed no Sox9 inactivation (0% recombination efficiency), while mutant animals showed varying degrees of Sox9 deletion. No animals were excluded from the analysis based on health status, tissue quality, or other technical reasons. The classification of mutant retinal	X

	phenotypes into "mild" or "extreme" categories was based on the qualitative assessment of outer nuclear layer (ONL) integrity, as detailed in Results and Supplementary File 1A, but all mutant animals were included in the analyses regardless of phenotypic severity.	

**Sample definition and in-laboratory replication**	**Indicate where provided: section/figure legend**	**N/A**
State number of times the experiment was replicated in the laboratory.	Material and Methods; Figure legends; Supplementary File 1.<br><br>All experiments were conducted using multiple independent biological replicates (individual mice). Key experimental findings were validated across animals analyzed at various time points after tamoxifen administration (ranging from 10 to 365 days). For example, retinal analysis included 7 control mice and 24 Sox9Δ/Δ mice, while corneal LacZ analysis included 4 control mice and 5 mutant mice. Sample sizes for each experiment are specified in figure legends and Supplementary File 1.	
Define whether data describe technical or biological replicates.	Material and Methods; Figure legends; Supplementary File 1:<br><br>All data presented represent **biological replicates**, where each individual mouse constitutes an independent biological replicate. For certain quantitative measurements (e.g., SOX8/SOX9 cell counting, Table S2), technical replicates were performed by conducting two independent counts on tissue sections from the same animal to ensure measurement accuracy. For histological analyses, both eyes from each mouse were examined to confirm consistency between paired samples, but the animal was considered the unit of biological replication.	

Ethics	Indicate where provided: section/submission form	N/A
Studies involving human participants: State details of authority granting ethics approval (IRB or equivalent committee(s), provide reference number for approval.	Material and Methods, final paragraph:<br><br>All animal procedures and housing conditions were approved by the University of Granada Ethics Committee for Animal Experimentation and the Consejería de Agricultura, Ganadería, Pesca y Desarrollo Sostenible of the Andalusian government, Junta de Andalucía (protocol reference: 12/12/2016/177). Mice were housed under Specific Pathogen-Free (SPF) conditions at the Center for Biomedical Research, University of Granada, Spain, with ad libitum access to food and water, enrichment (nesting materials and hiding places), temperature 22 ± 2°C, humidity 55 ± 10%, and a 12-hour dark/light cycle.	
Studies involving experimental animals: State details of authority granting ethics approval (IRB or equivalent committee(s), provide reference number for approval.	Material and Methods, final paragraph:<br><br>All animal procedures were approved by the University of Granada Ethics Committee for Animal Experimentation and the Consejería de Agricultura, Ganadería, Pesca y Desarrollo Sostenible of the Andalusian government, Junta de Andalucía (protocol reference: 12/12/2016/177). These authorities provided full ethical oversight for all experimental procedures.	
Studies involving specimen and field samples: State if relevant permits obtained, provide details of authority approving study; if none were required, explain why.	No field-collected samples or wild specimens were used in this study. All specimens analyzed were derived from laboratory-bred mice housed under institutional animal care protocols. No collection permits or field sampling approvals were required.	X

Dual Use Research of Concern (DURC)	Indicate where provided: section/submission form	N/A
If study is subject to dual use research of concern regulations, state the authority granting approval and reference number for	This study did not involve any activities subject to dual use	X

the regulatory approval.	research of concern regulations. No regulatory approval was required.	

Analysis:

**Attrition**	**Indicate where provided: section/figure legend**	**N/A**
Describe whether exclusion criteria were pre-established. Report if sample or data points were omitted from analysis. If yes, report if this was due to attrition or intentional exclusion and provide justification.	Results; Supplementary File 1: No pre-established exclusion criteria were applied in this study. All mice that received tamoxifen treatment were included in the analysis regardless of the degree of Sox9 recombination efficiency or phenotypic severity. Control animals (Cre-negative Sox9^flox/flox^, n=7) and mutant animals (CAGG-CreER^TM^;Sox9^flox/flox^, n=24) all received tamoxifen treatment. No animals were excluded due to health issues, technical problems, or as outliers. Sample sizes (n) for each experimental group are reported in figure legends and Supplementary File 1, representing all animals analyzed without any exclusions. The variability in phenotypic outcomes among mutants reflects the mosaic nature of tamoxifen-induced Cre-mediated recombination and was intentionally captured in the analyses.	X

**Statistics**	Indicate where provided: section/figure legend	**N/A**
Describe statistical tests used and justify choice of tests.	Material and Methods, section "Statistical analysis"; Supplementary File 1: Statistical analyses were performed using appropriate tests selected based on data distribution and sample characteristics. Normality was assessed using the Shapiro-Wilk test, and homogeneity of variances was evaluated using Levene's test. For comparisons between two groups, when assumptions of normality and homoscedasticity were met, Student's t-test (2-sample T-test) was used. When these assumptions were violated, the	

	non-parametric Mann-Whitney U test was applied. For multiple group comparisons, the Kruskal-Wallis test with post-hoc Dunn's test was employed when parametric assumptions were not satisfied. For count data with an excess of zeros (e.g., TUNEL-positive cell counts), a zero-inflated Poisson model was used to account for the inflated frequency of zero values characteristic of transient apoptotic events. All statistical details, including test statistics, degrees of freedom, and p-values, are provided in the Supplementary File 1. Significance was set at $p < 0.05$ for all analyses.	

**Data availability**	**Indicate where provided: section/submission form**	**N/A**
For newly created and reused datasets, the manuscript includes a data availability statement that provides details for access (or notes restrictions on access).	No newly generated high-throughput datasets from this study have been deposited in public repositories. All quantitative data are provided in the Supplementary File 1.	X
When newly created datasets are publicly available, provide accession number in repository OR DOI and licensing details where available.	No newly generated high-throughput datasets (e.g., sequencing data, imaging datasets) from this study have been deposited in public repositories. All quantitative data supporting the findings are provided in Supplementary File 1 included with the manuscript. Raw image data and additional materials are available from the corresponding authors upon reasonable request.	X
If reused data is publicly available provide accession number in repository OR DOI, OR URL, OR citation.	Material and Methods, section "Single-cell RNA-sequencing dataset analyses": The study utilized publicly available scRNA-seq data obtained from the Gene Expression Omnibus (GEO) repository under accession number GSE167992 (Altshuler et al., 2021, Nature Communications, DOI: 10.1038/s41467-021-23945-1).	

**Code availability**	**Indicate where provided: section/figure legend**	**N/A**

For any computer code/software/mathematical algorithms essential for replicating the main findings of the study, whether newly generated or re-used, the manuscript includes a data availability statement that provides details for access or notes restrictions.	Material and Methods, sections "Single-cell RNA-sequencing dataset analyses" and "Statistical analysis": The manuscript describes all software and computational tools used for data analysis. scRNA-seq analyses were performed using publicly available software: Seurat version 5.0.3 (R package), Python Scanpy suite, and clusterProfiler (R package). Statistical analyses were conducted using R and Python scipy.stats module. No custom code or novel algorithms were developed for this study; all analyses used standard, publicly available packages with default or commonly used parameters as described in the Methods section.	
Where newly generated code is publicly available, provide accession number in repository, OR DOI OR URL and licensing details where available. State any restrictions on code availability or accessibility.	No custom code or novel computational algorithms were generated for this study. All analyses used standard, publicly available software packages.	X
If reused code is publicly available provide accession number in repository OR DOI OR URL, OR citation.	Material and Methods, sections "Single-cell RNA-sequencing dataset analyses" and "Statistical analysis": All software used is publicly available: Seurat (https://satijalab.org/seurat/), Scanpy (https://scanpy.readthedocs.io/), clusterProfiler (Bioconductor), R statistical software (https://www.r-project.org/), and Python scipy.stats (https://scipy.org/). Citations for these packages are provided in the reference list.	

Reporting:

The MDAR framework recommends adoption of discipline-specific guidelines, established and endorsed through community initiatives.

Adherence to community standards	Indicate where provided: section/figure legend	N/A
State if relevant guidelines (e.g., ICMJE, MIBBI, ARRIVE, STRANGE) have been followed, and whether a checklist (e.g., CONSORT, PRISMA, ARRIVE) is provided with the manuscript.	Material and Methods; MDAR checklist (this document): This study follows the ARRIVE (Animal Research: Reporting of In Vivo Experiments) guidelines for reporting animal research. The manuscript provides comprehensive details on animal husbandry, experimental design, sample sizes, statistical methods, and ethical approval as recommended by	

	ARRIVE 2.0 guidelines. Additionally, the study adheres to eLife's MDAR (Materials, Design, Analysis, and Reporting) framework requirements for transparent reporting in life sciences research. The completed MDAR checklist is provided as part of the submission documentation.	

* We provide the following guidance regarding transparent reporting and statistics; we also refer authors to Ten common statistical mistakes to watch out for when writing or reviewing a manuscript.

Sample-size estimation

- You should state whether an appropriate sample size was computed when the study was being designed
- You should state the statistical method of sample size computation and any required assumptions
- If no explicit power analysis was used, you should describe how you decided what sample (replicate) size (number) to use

Replicates

- You should report how often each experiment was performed
- You should include a definition of biological versus technical replication
- The data obtained should be provided and sufficient information should be provided to indicate the number of independent biological and/or technical replicates
- If you encountered any outliers, you should describe how these were handled
- Criteria for exclusion/inclusion of data should be clearly stated
- High-throughput sequence data should be uploaded before submission, with a private link for reviewers provided (these are available from both GEO and ArrayExpress)

Statistical reporting

- Statistical analysis methods should be described and justified
- Raw data should be presented in figures whenever informative to do so (typically when N per group is less than 10)
- For each experiment, you should identify the statistical tests used, exact values of N, definitions of center, methods of multiple test correction, and dispersion and precision measures (e.g., mean, median, SD, SEM, confidence intervals; and, for the major substantive results, a measure of effect size (e.g., Pearson's r, Cohen's d)
- Report exact p-values wherever possible alongside the summary statistics and 95% confidence intervals. These should be reported for all key questions and not only when the p-value is less than 0.05.

Group allocation

- Indicate how samples were allocated into experimental groups (in the case of clinical

studies, please specify allocation to treatment method); if randomization was used, please also state if restricted randomization was applied

- Indicate if masking was used during group allocation, data collection and/or data analysis
